# Supplementary material for: Combining evidence from human genetic and functional screens to identify pathways altering obesity and fat distribution
Source: Am J Hum Genet. 2025 Sep 4;112(10):2316–37. doi: 10.1016/j.ajhg.2025.08.013 (PMC12696503; doi:10.1016/j.ajhg.2025.08.013)
Supplement: Document S1. Figures S1–S9, Tables S1, S2, S9–S14, and Notes S1 and S2 [file mmc1.pdf]

The American Journal of Human Genetics, Volume *112*

Supplemental information

Combining evidence from human genetic and functional screens to identify pathways altering obesity and fat distribution

Nikolas A. Baya, Ilknur Sur Erdem, Samvida S. Venkatesh, Saskia Reibe, Philip D. Charles, Elena Navarro-Guerrero, Barney Hill, Frederik H. Lassen, Melina Claussnitzer, Duncan S. Palmer, and Cecilia M. Lindgren

Supplemental notes

Supp. Note 1. Calculating unweighted burden effect sizes

First, summary statistics for variant-level results are recalculated relative to the minor allele: the sign of the t-statistic is flipped if the original effect allele is not the minor allele and the frequency of the effect allele is recalculated accordingly. Then, for all single-variant and grouped ultra-rare variant burden results in a given gene which satisfy the maximum minor allele frequency and variant consequence of the desired result to unweight, the following summary statistics are summed: t-statistic, variance of t-statistic, and MAF. The unweighted t-statistic is calculated by dividing the summed t-statistics by the summed variance of t-statistics. The unweighted MAF is the summed MAF across variants and grouped ultra-rare variants in the gene.

$$t_{\text{unweighted,gene}} = \frac{\sum_j t_{\text{weighted},j}}{\sum_j \text{Var}(t_{\text{weighted},j})}, \text{for variant } j \text{ in } gene \quad (3)$$

$$\text{MAF}_{\text{unweighted,gene}} = \sum_j \text{MAF}_{\text{weighted},j}, \text{for variant } j \text{ in } gene \quad (4)$$

Supp. Note 2. Significant genes with low minor allele count

In sex-combined and sex differential gene-level associations we sought to avoid spurious associations by excluding putatively significant genes if they were supported by a total MAC < 10 across both sexes.

Among the sex-combined gene-level associations which were significant at FDR $\leq 1\%$ (SKAT-O $P \leq 4.37 \times 10^{-5}$), there were seven genes which were excluded for having MAC < 5: *DEFB112* (MAC=1), *CHMP4B* (MAC = 2), *FEZF2* (MAC = 3), *GLP1R* (MAC = 3), *PCBD2* (MAC = 4), *VGF* (MAC = 8), and *TM4SF20* (MAC = 9) (Table S3).

In the sex differential analysis, we excluded a significant (sex-difference $P < 2.67 \times 10^{-6}$) differential effect at *SENP5* for visceral adipose tissue volume (β_{female} (SE) = 0.08930 (0.028055), $\beta_{\text{male}} = -0.09967$ (0.028675), $P_{\text{diff}} = 2.33 \times 10^{-6}$) due to combined MAC < 10 ($\text{MAC}_{\text{female}} = 2$, $\text{MAC}_{\text{male}} = 2$) (Table S5).

Supplemental figures

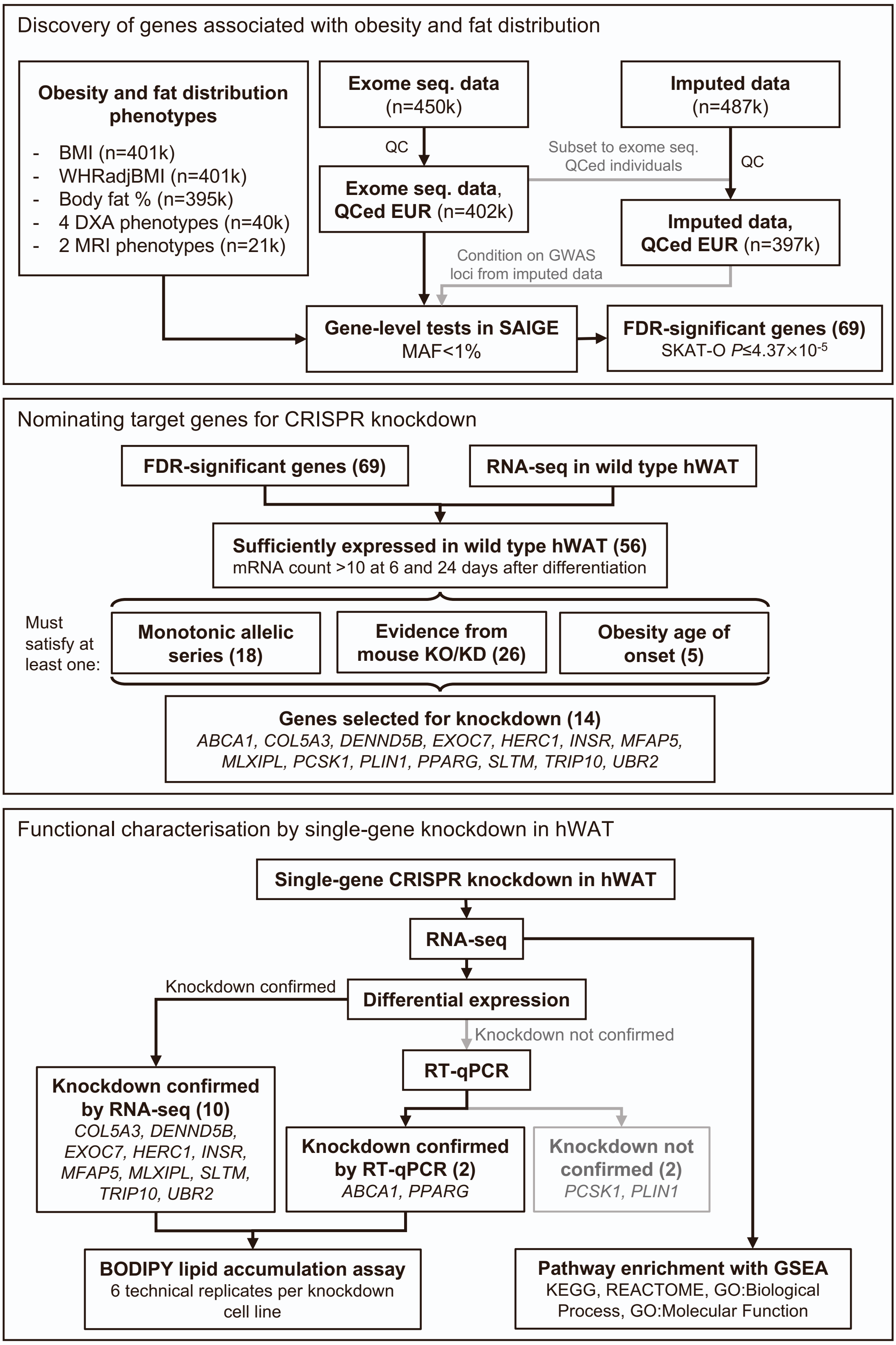

Figure S1: Study design.

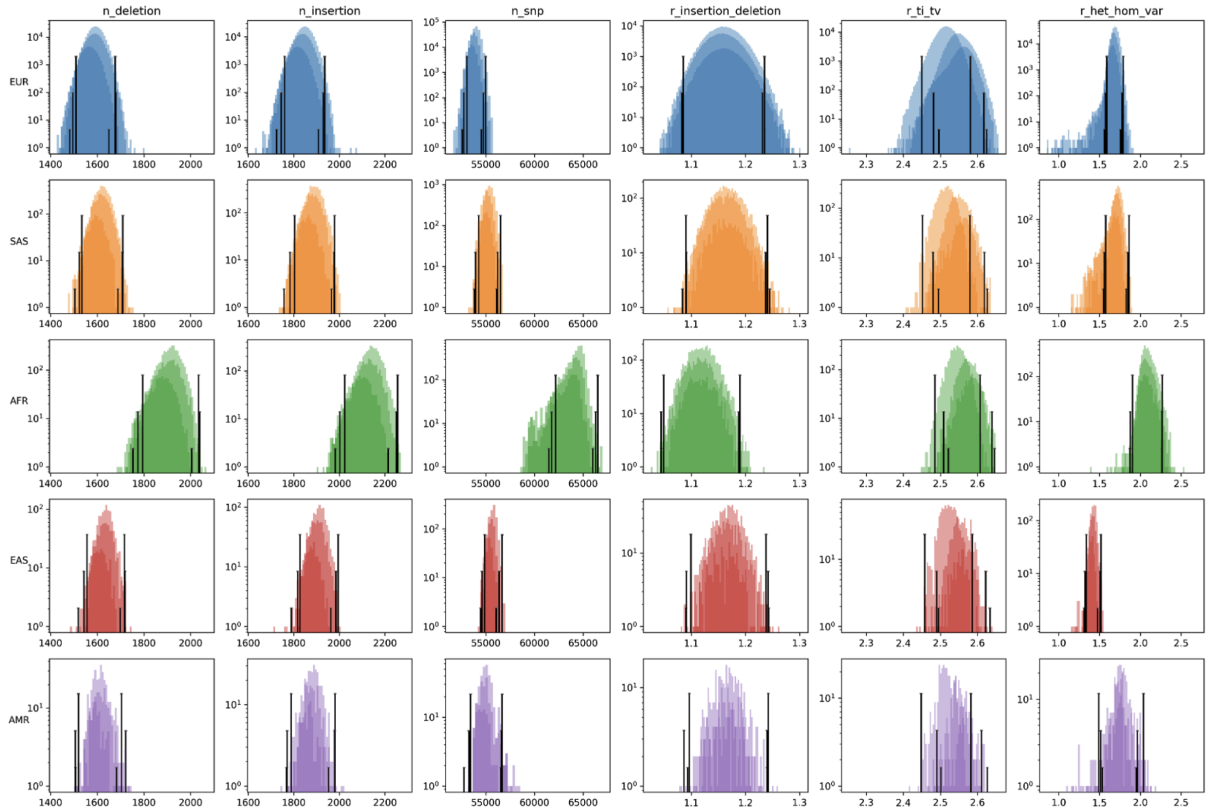

Figure S2: MAD thresholds, split by tranche. Samples with any of `n_deletion`, `n_insertion`, `n_snp`, `r_insertion_deletion`, `r_ti_tv`, and `r_het_hom_var` exceeding four MADs from the median are removed. MAD thresholds are displayed as vertical lines, conditional on tranche size (50k, 200k, 250k).

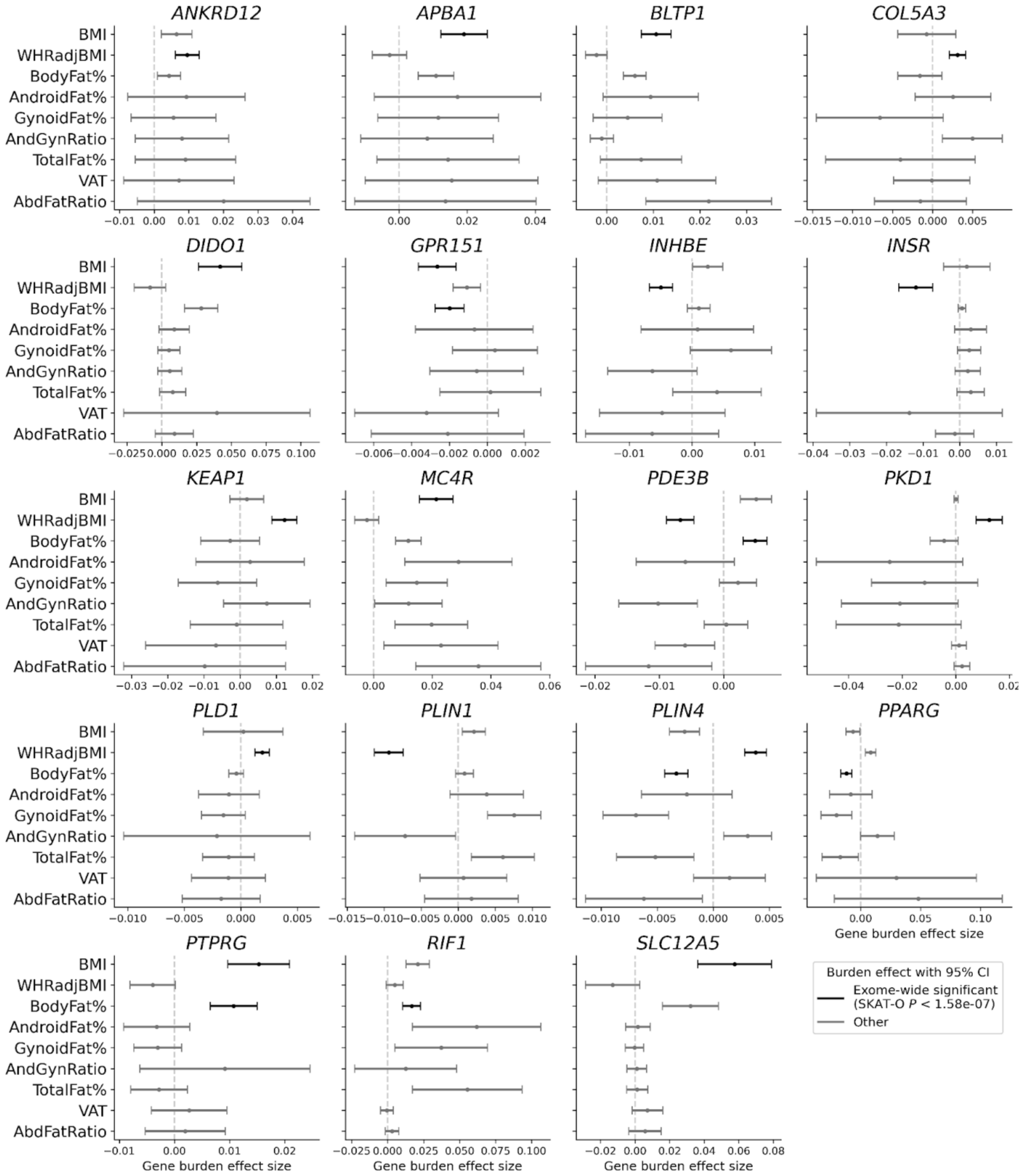

Figure S3: Gene burden effects across all nine obesity and fat distribution traits for 19 genes with exome-wide significant burden associations. Confidence intervals for effect size defined as ±1.96 standard errors. Only the result of the consequence mask with the lowest SKAT-O P-value is shown for each trait-gene pair. The vertical dotted line marks an effect size of zero. VAT, visceral adipose tissue.

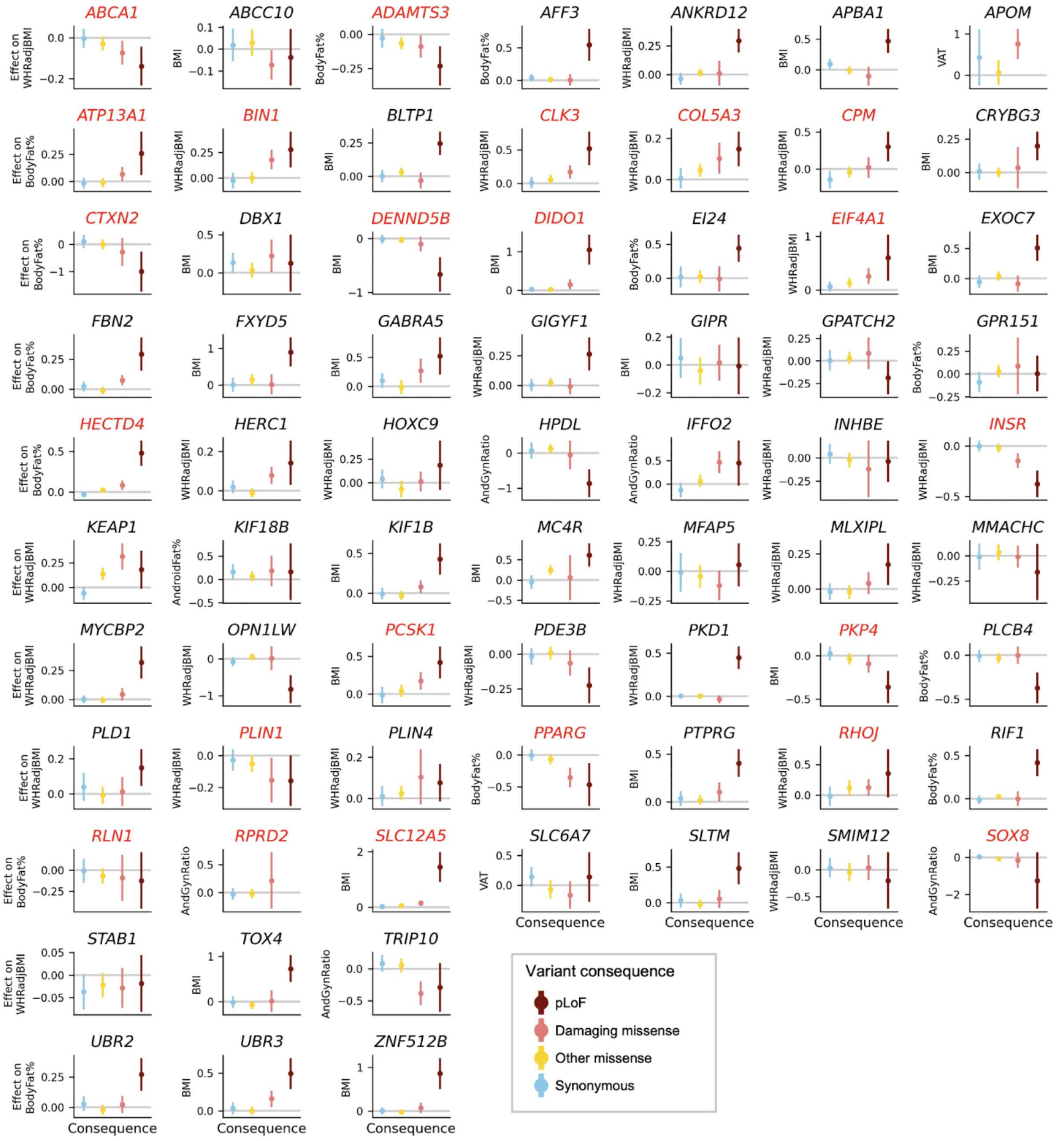

Figure S4: Ultra-rare variant allelic series for all FDR≤1% significant genes. Monotonic allelic series are highlighted with red gene symbols. Allelic series are defined as monotonic if they have strictly increasing or strictly decreasing point estimates of effect size across increasingly deleterious variant consequences (least to most deleterious: synonymous, other missense, damaging missense, pLoF). Effect sizes come from ultra-rare variant burden for variants with minor allele count ≤ 10 in each gene, grouped by variant consequence. Confidence intervals for effect size defined as ±1.96 standard errors. pLoF, predicted loss-of-function.

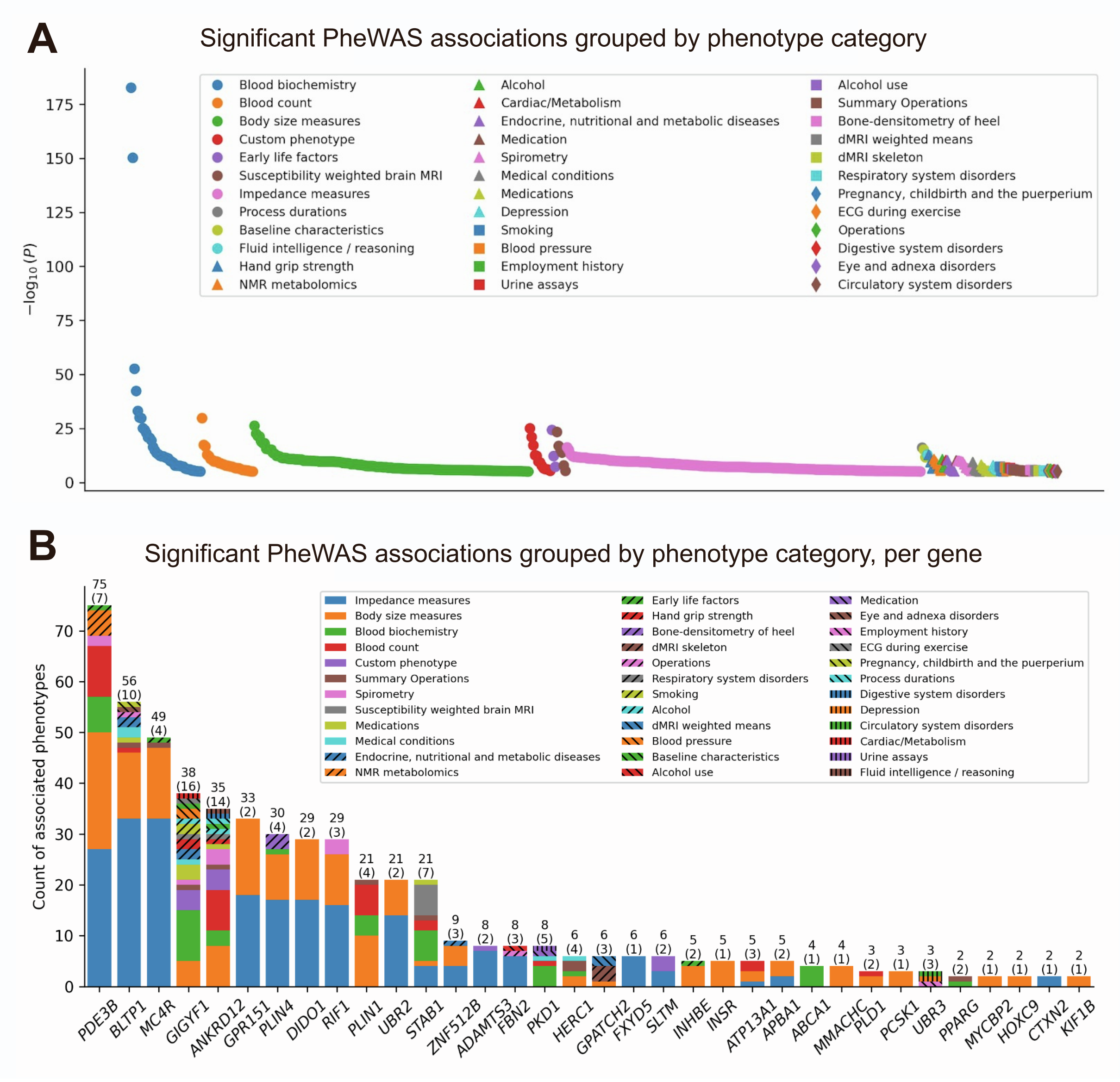

Figure S5: PheWAS of obesity and fat distribution associated genes using Genebass summary statistics. Only the pLoF variant mask results from Genebass are used. Significant associations are controlled for FDR$\leq$ 1% (SKAT-O $P \leq 9.98 \times 10^{-6}$), resulting in 549 significant associations across 211 phenotypes and 41/69 obesity and fat distribution associated genes. **A**, Significant associations grouped by phenotype category, with phenotype groups ordered from left to right by the lowest P-value in the category. Significance of association is measured on the y-axis as $-\log 10$(Genebass SKAT-O P-value). **B**, Significant associations per gene, grouped by phenotype category. The total number of significant phenotype associations is shown at the top of each bar, with the number of phenotype categories shown in parentheses. Only genes with at least two phenotype associations are shown.

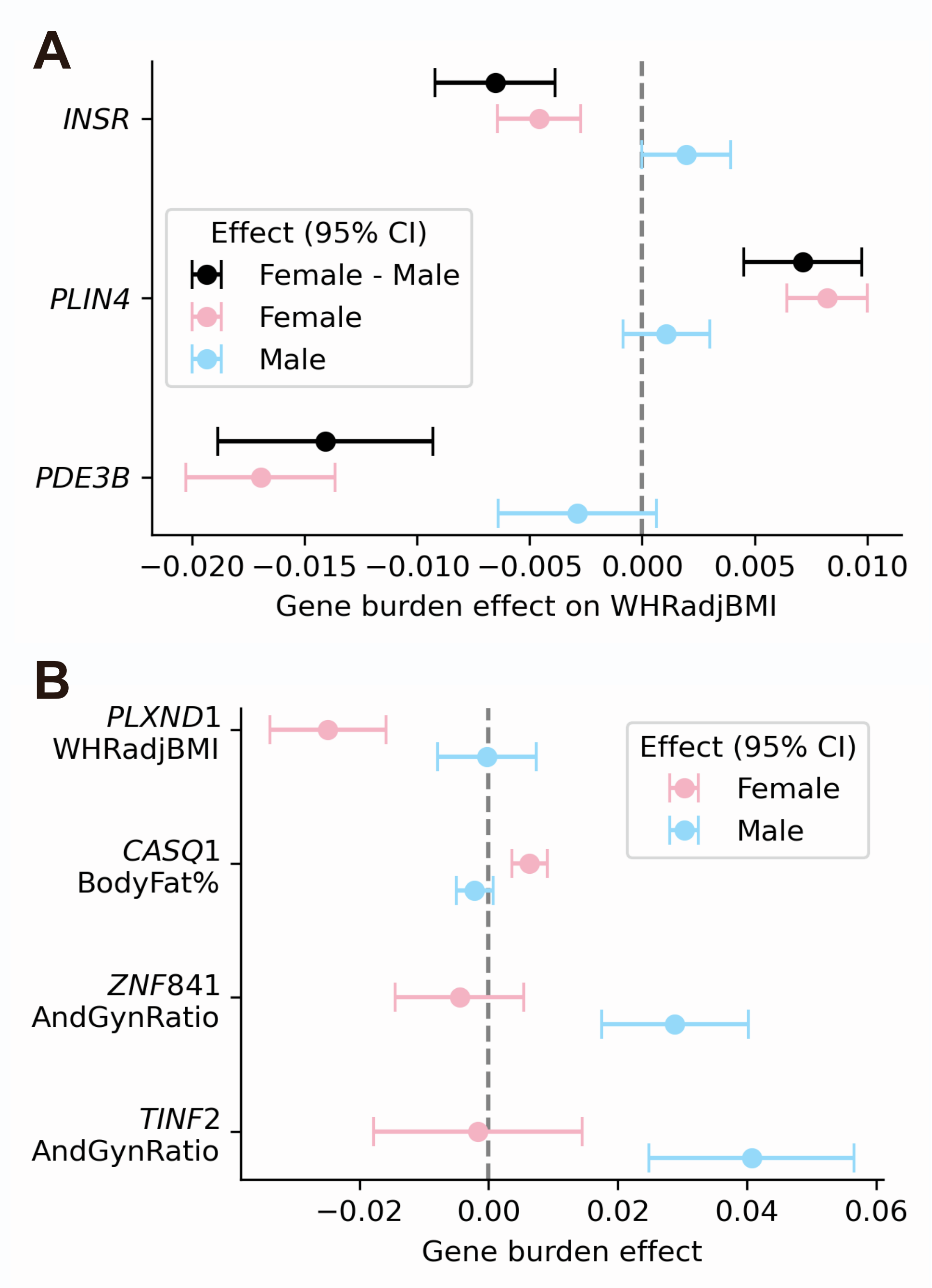

Figure S6: Sex-differential and sex-specific analysis. **A**, Genes with significant sex-differential effects (sex-difference $P < 2.67 \times 10^{-6}$, Bonferroni adjusted for 18,737 genes tested for sex-differential effects). All three significant sex-differential gene burden effects are on WHRadjBMI. **B**, Female- (*PLXND1*, *CASQ1*) and male-specific (*ZNF841*, *TINF2*) gene-level significant associations ($P < 2.67 \times 10^{-6}$, Bonferroni adjusted for 18,737 genes tested for sex-specific effects). CIs for effect size defined as ±1.96 standard errors.

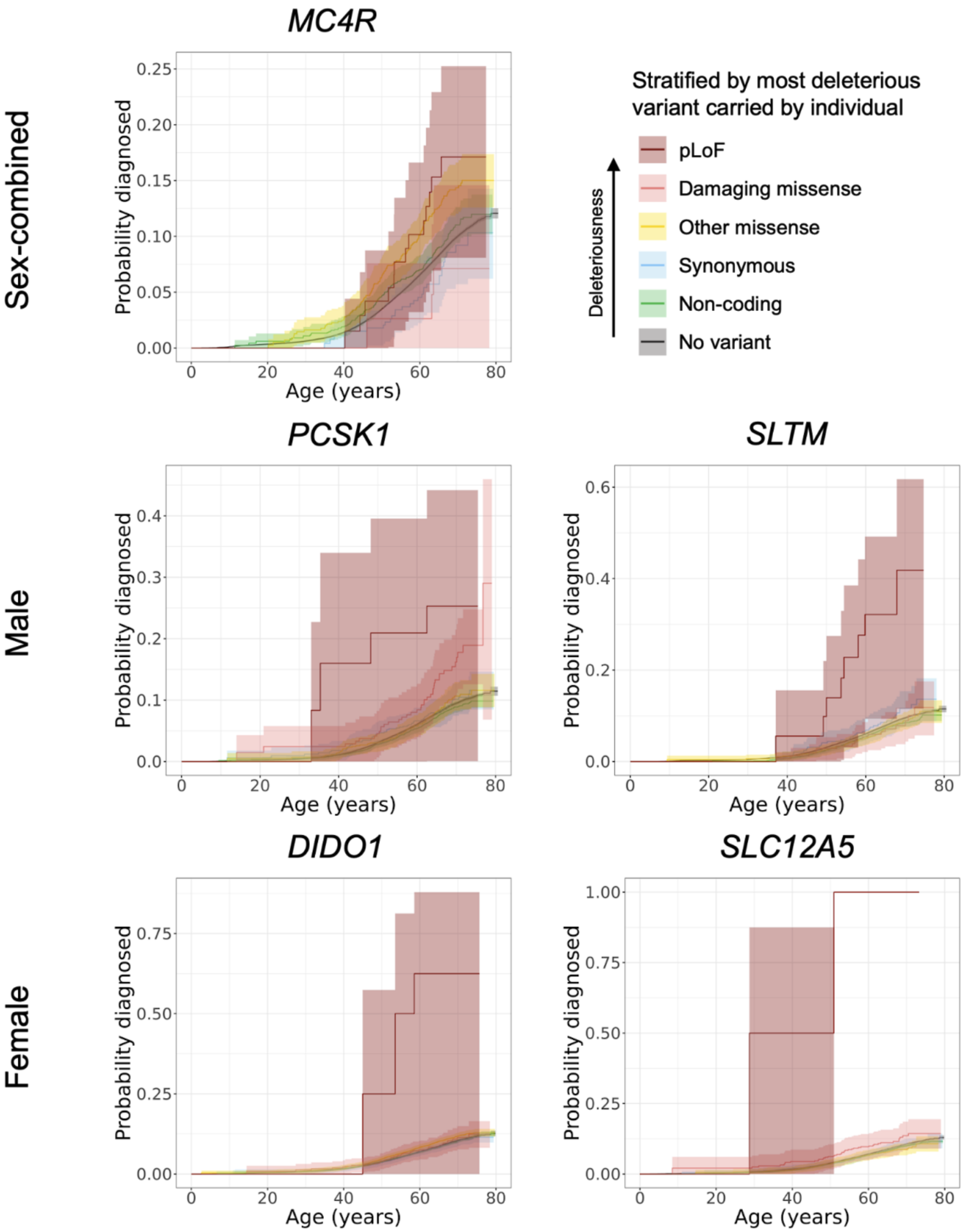

Figure S7: Longitudinal obesity age-at-onset analysis. Longitudinal analysis was performed using Cox-proportional hazards modelling (see "Age at diagnosis longitudinal analyses" methods). Individuals are stratified by the most deleterious variant carried in the gene. Only genes with significant age-of-onset associations in both sexes (*MC4R*), males only (*PCSK1*, *SLTM*), or females only (*DIDO1*, *SLC12A5*) are shown. 95% confidence intervals are indicated by shading.

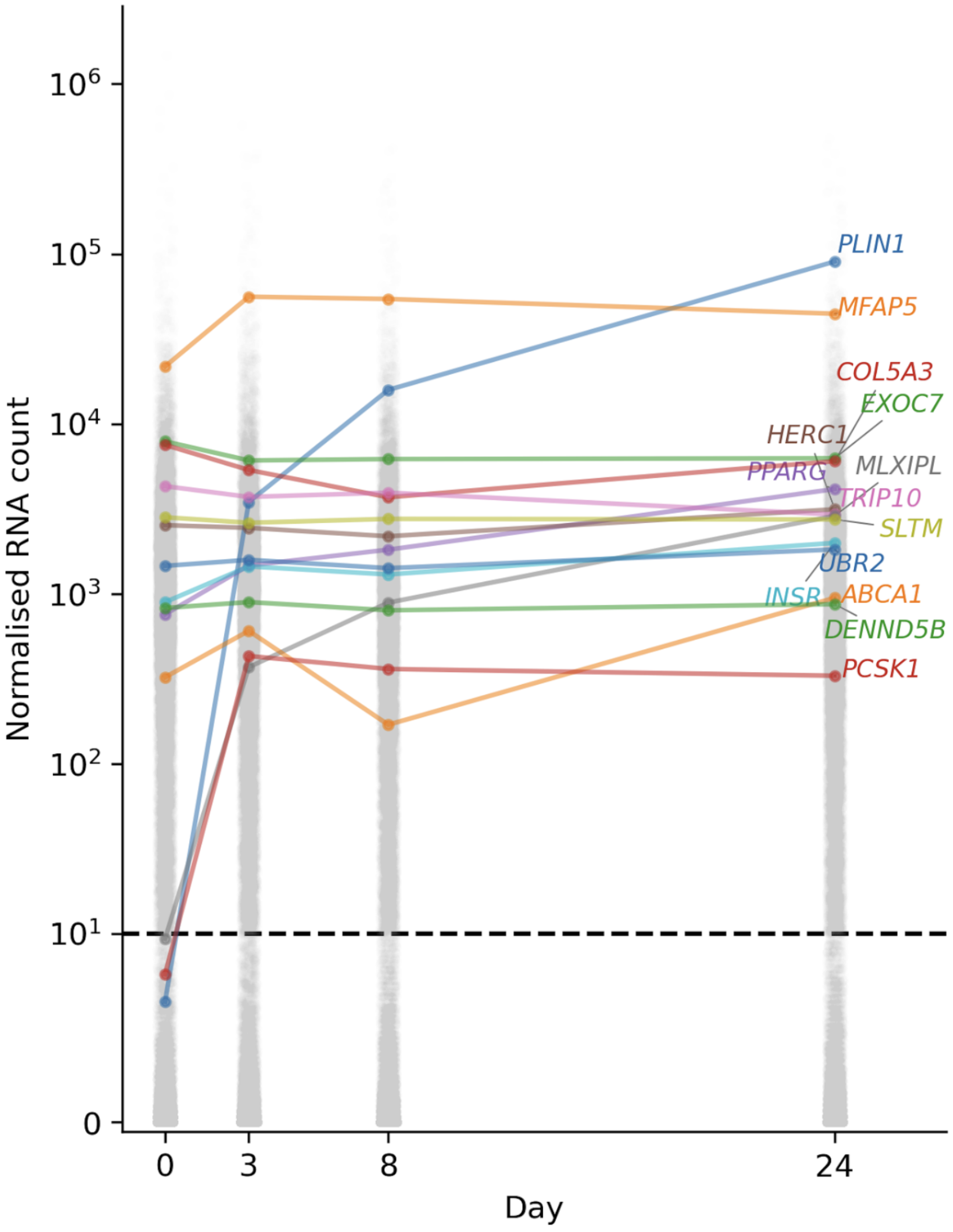

Figure S8: Longitudinal mRNA expression in wild type human white adipose tissue of genes selected for knockdown. Normalised mRNA count was measured at four time points across 24 days of differentiation. Day 0 corresponds to the undifferentiated state. Coloured traces are genes selected for knockdown. All other genes are indicated with grey points. The horizontal dashed line indicates the minimum threshold needed on days 8 and 24 for a gene to be selected as a knockdown target. The *y*-axis uses the 'symlog' scale, such that the scale is linear between 0 and 10 and logarithmic for values greater than 10.

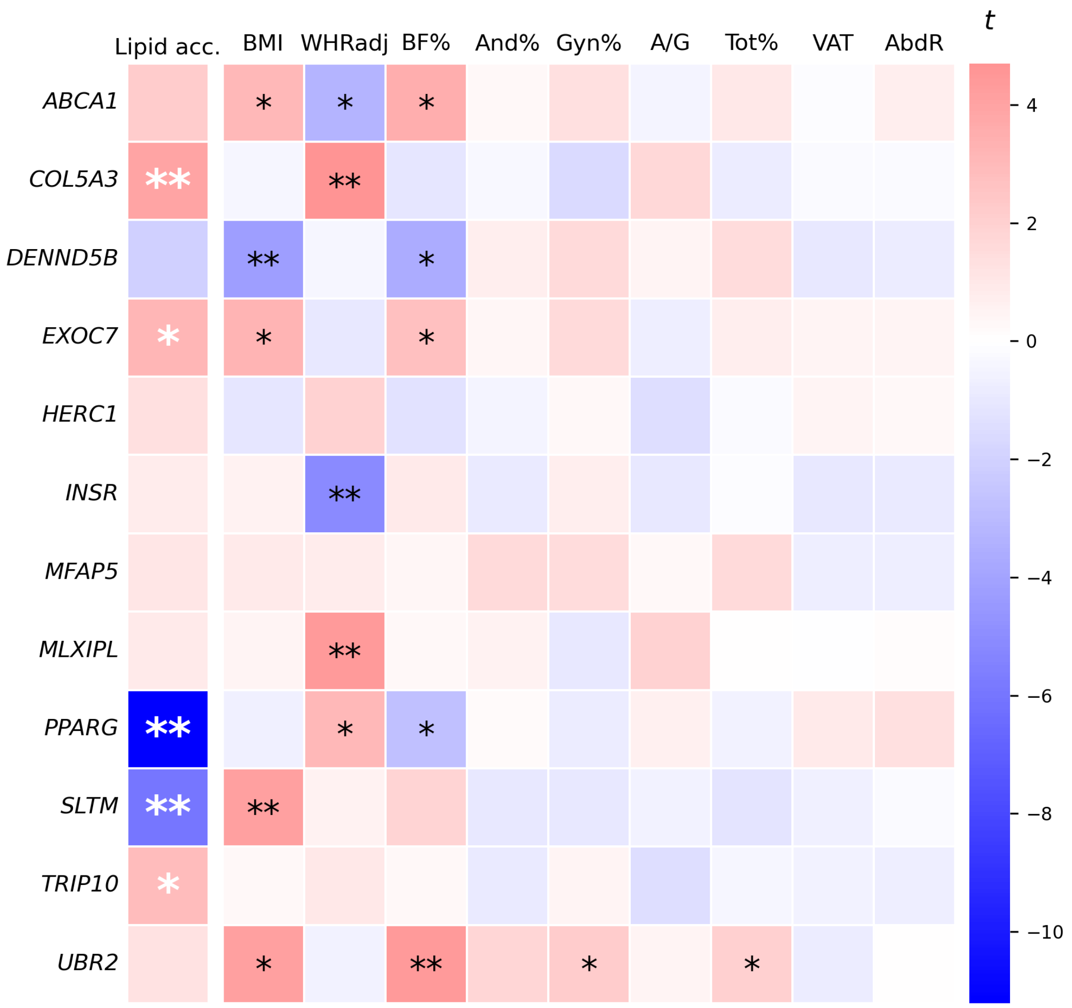

Figure S9: Comparison of lipid accumulation assay results and gene burden across traits. Both heatmaps are coloured according to t-statistics and share the same colour bar. Left single-column heatmap indicates effect of gene knockdown on lipid accumulation in hWAT. Two-sided t-test of mean FC relative to Cas9-empty cells: $**P < 0.05/14$, $*P < 0.05$, using white asterisks. Right multi-column heatmap indicates gene burden effects for each gene-trait pair. Only results from the pLoF consequence mask are used for each gene-trait pair, with the intention of modelling the equivalent of a human knockdown. Significance of burden test: ***Exome-wide significant ($P < 1.58 \times 10^{-7}$), **FDR$\leq$ 1% significant ($P \leq 4.37 \times 10^{-5}$), *Nominal significance ($P < 0.05$), using black asterisks. WHRadj, waist-to-hip ratio adjusted for BMI. BF%, body fat percentage. And%, android fat percentage. Gyn%, gynoid fat percentage. A/G, android-gynoid fat percentage ratio. Tot%, total fat percentage. VAT, visceral adipose tissue. AbdR, abdominal fat ratio.

Supplemental tables

Table S1: Sample removal based on sample outliers defined by MAD thresholds. Threshold = median ±4 MADs, split by sequencing tranche.

QC Metric	# Samples in UK Biobank Whole Exome Sequencing Tranche		
	250k	150k	50k
**Total before QC**	**237,091**	**139,745**	**46,034**
Number of deletions	1,510	825	344
Number of insertions	1573	1,118	236
Number of SNPs	1,856	1,013	362
Ratio of insertions to deletions	1,741	954	339
Ratio of transitions to transversions	2,890	1,598	1781
Ratio of heterozygous variants to homozygous alternate variants	2,301	1,496	478
Total failing	10,861	6,396	3,238
**Total passed QC**	**226,230**	**133,349**	**42,796**
**Grand total**			**402,375**

Table S2: Sample sizes and phenotype SDs for each adiposity-related trait (sex-combined and sex-stratified). Waist-to-hip ratio (WHR) is calculated by dividing waist circumference (UKB Field ID: 48) by hip circumference (UKB Field ID: 49). Adjustment for BMI was calculated by regressing out BMI (UKB Field ID: 21001) from WHR. Android-gynoid tissue fat percentage ratio is calculated by dividing android tissue fat percentage by gynoid tissue fat percentage.

Phenotype	UKB Field ID	Both sexes		Female		Male	
		N	Std. dev.	N	Std. dev.	N	Std. dev.
Body mass index (BMI)	21001	401059	4.74783	217716	5.11381	183343	4.22661
Waist-to-hip ratio, adjusted for BMI (WHRadjBMI)	n/a	400948	0.08064	217655	0.06263	183293	0.05214
Body fat percentage (BF%)	23099	395147	8.50612	214846	6.86752	180301	5.80123
Android tissue fat percentage	23247	39671	0.11148	20456	0.11721	19215	0.10322
Gynoid tissue fat percentage	23264	39671	0.09288	20456	0.06664	19215	0.06159
Android-gynoid tissue fat percentage ratio	n/a	39671	0.08305	20456	0.07372	19215	0.06631
Total tissue fat percentage	23281	39671	0.28970	20456	0.20838	19215	0.24515
Visceral adipose tissue (VAT) volume	22407	21253	2.28788	11000	1.52205	10253	2.35928
Abdominal fat ratio	22434	20687	0.11222	10892	0.10543	9795	0.09907

Table S9: Guide RNAs per gene target.

Gene	Sequence	Target site	Guide score	Strand
ABCA1	TGGCTTGTTGGCCTCAGCTG	chr9:107665940-107665959	1.292	-
ABCA1	GAGTCGGGTAACGGAAACAG	chr9:107646751-107646770	2.216	+
ABCA1	CCAGAAAGACACCAGCATGA	chr9:107645374-107645393	1.298	-
ABCA1	GAAGCTTCAAGATTTCCTGG	chr9:107624058-107624077	3.024	-
COL5A3	AAGGCCCTGGGTGTGCAGGG	chr19:10116871-10116890	2.47	-
COL5A3	TAGCTGGACTCACTGTGCTG	chr19:10116267-10116286	1.452	-
COL5A3	AAGAAAGGGCGAGGTCGCAA	chr19:10114311-10114330	1.961	-
COL5A3	TCACCTAGGAGACCCAGCGC	chr19:10116517-10116536	2.049	+
DENND5B	GATCGACGCGGACAGCGGGC	chr12:31743661-31743680	1.511	-
DENND5B	GTCAAGGCAAATATGAGCAG	chr12:31595765-31595784	2.702	-
DENND5B	GGAGCTCAAATGCCTCCCGA	chr12:31632595-31632614	3.414	+
DENND5B	GTGTCAAAGACCCGAAGCAA	chr12:31600561-31600580	1.451	+
EXOC7	AGCAGACGGAGAATCTGCAG	chr17:74097858-74097877	1.881	-
EXOC7	AAGCATGGCCAAGATTCAGA	chr17:74097408-74097427	1.032	-
EXOC7	TGAGCGCGGGAAGGAGGCCC	chr17:74094069-74094088	1.485	-
EXOC7	AGGAGGCATCCGCTCGACGG	chr17:74099741-74099760	2.321	-
HERC1	AGTGCATCAGTGCTTGACCG	chr15:64067353-64067372	2.751	+
HERC1	GAACCAACCAGAACATCGGA	chr15:64056341-64056360	1.698	-
HERC1	AGAAGGAGAAGTCTTCAGTT	chr15:64048725-64048744	1.251	-
HERC1	AATGTAGGAGAGGTTTCTTG	chr15:64046775-64046794	1.041	-
INSR	AACCTGATGAACATCACCCG	chr19:7267586-7267605	3.45	-
INSR	ACAAATGCAAGAACTCGCGG	chr19:7184410-7184429	3.852	-
INSR	GAGGTGGCACACCTTGGGAC	chr19:7174694-7174713	1.742	+
INSR	GGAAAGTGACACCAGAGCGT	chr19:7172348-7172367	2.616	+
MFAP5	TATGTCGCTCTTGGGACCCA	chr12:8814682-8814701	1.055	-
MFAP5	GAACTCACCCAAGTCATCTG	chr12:8807025-8807044	1.06	+
MFAP5	GTCGGAAGTAATTGGAGCGA	chr12:8800734-8800753	1.69	+
MFAP5	GAGCGACGGAGTCTCCTAGG	chr12:8800748-8800767	2.018	+
MLXIPL	GGACTCGGACACAGACTCGG	chr7:73038735-73038754	1.755	-
MLXIPL	CCTGAACAACGCCATCTGGA	chr7:73030419-73030438	0.933	-
MLXIPL	TGATGCGCACCGGAAGCCGG	chr7:73021922-73021941	2.232	-
MLXIPL	CTGGAAGCGGCGCATCGAGG	chr7:73021708-73021727	1.292	-
PCSK1	AGCAGCCTCGGCCATCGCCG	chr5:95768596-95768615	2.973	-
PCSK1	GAAGTAAACGTTCAGCTCTA	chr5:95761581-95761600	1.149	-
PCSK1	ATGAAGGTCCAGCTTGGGCA	chr5:95759119-95759138	1.025	+
PCSK1	AATGCTGGATGGCATTGTGA	chr5:95748168-95748187	1.061	-
PLIN1	GCGAGGATGGCAGTCAACAA	chr15:90220707-90220726	1.973	-
PLIN1	GAAGACCTACACCAGCACTA	chr15:90216558-90216577	3.053	-
PLIN1	CCGAGGCTTGGACCACCTGG	chr15:90214751-90214770	0.934	-
PLIN1	CTTGTCTGAAGTGCTCGCGA	chr15:90213386-90213405	1.757	+
PPARG	ATTCACAAGAACAGATCCAG	chr3:12421366-12421385	1.553	+
PPARG	AGAGCCTTCCAACTCCCTCA	chr3:12422894-12422913	1.344	+
PPARG	GTTTCAGAAATGCCTTGCAG	chr3:12434214-12434233	1.895	+
PPARG	GCTGACCAAAGCAAAGGCGA	chr3:12447526-12447545	3.041	+
SLTM	TGAAGTCCGAGCTGAAGCGG	chr15:59225657-59225676	2.418	-
SLTM	CAAGAGGCACATGAGCAAGA	chr15:59205858-59205877	1.651	-
SLTM	TAAGAAGGAAGACTGCGTGA	chr15:59191789-59191808	3.514	-
SLTM	GACGAGCAATGGAACTTCGA	chr15:59185098-59185117	0.893	-
TRIP10	CTTCGGCCATGTGACTCCGA	chr19:6743739-6743758	1.174	-
TRIP10	TGCTCGAGCGCCACACGCAG	chr19:6741034-6741053	0.851	+
TRIP10	CAAGACATACACGGACACTG	chr19:6743062-6743081	1.581	-
TRIP10	ATTTGAGCGGGACTGCCGGG	chr19:6743516-6743535	2.732	+
UBR2	TTCGGCCGAGGAGATTGCGG	chr6:42532114-42532133	3.19	+
UBR2	ATGCAGGATCTTCACCACAA	chr6:42541624-42541643	1.474	-
UBR2	TCCAAGATGACAACATCAGG	chr6:42561923-42561942	0.865	+
UBR2	TTGAGACATCGAGATGCCCA	chr6:42600353-42600372	1.808	+

Table S10: RNA-seq differential expression to confirm knockdown.

knockdown_cell_line	target_gene	ensembl_gene_id	log2fc	log2fc_se	zscore	pval_1sided	fc	fc_95ci_lower	fc_95ci_upper
ABCA1	ABCA1	ENSG00000165029	0.596	0.139	4.283	1.00e+00	1.511	1.251	1.825
COL5A3	COL5A3	ENSG00000080573	-0.197	0.088	-2.246	1.24e-02	0.872	0.774	0.983
DENND5B	DENND5B	ENSG00000170456	-1.789	0.127	-14.094	2.08e-45	0.289	0.243	0.344
EXOC7	EXOC7	ENSG00000182473	-0.698	0.051	-13.742	2.83e-43	0.617	0.575	0.661
HERC1	HERC1	ENSG00000103657	-1.216	0.186	-6.541	3.07e-11	0.431	0.335	0.554
INSR	INSR	ENSG00000171105	-0.940	0.105	-8.940	1.94e-19	0.521	0.452	0.601
MFAP5	MFAP5	ENSG00000197614	-1.364	0.138	-9.882	2.48e-23	0.388	0.322	0.469
MLXIPL	MLXIPL	ENSG00000009950	-2.455	0.295	-8.322	4.32e-17	0.182	0.122	0.272
PCSK1	PCSK1	ENSG00000175426	1.985	0.569	3.487	1.00e+00	3.959	1.827	8.580
PLIN1	PLIN1	ENSG00000166819	0.195	0.224	0.872	8.08e-01	1.145	0.845	1.553
PPARG	PPARG	ENSG00000132170	-0.165	0.128	-1.293	9.81e-02	0.892	0.750	1.061
SLTM	SLTM	ENSG00000137776	-1.139	0.061	-18.692	2.89e-78	0.454	0.418	0.493
TRIP10	TRIP10	ENSG00000125733	-0.481	0.063	-7.668	8.74e-15	0.717	0.658	0.780
UBR2	UBR2	ENSG00000024048	-0.521	0.096	-5.441	2.65e-08	0.697	0.612	0.794

Table S11: PCR primers.

Gene/isoform	Forward/Reverse	Sequence
PPARG1	Forward	AAAGAAGCCGACACTAAACC
PPARG1	Reverse	CTTCCATTACGGAGAGATCC
PPARG2	Forward	TCCATGCTGTTATGGGTGAA
PPARG2	Reverse	TCAAAGGAGTGGGAGTTGTC
ACTIN	Forward	CCAACCGCGAGAAGATGA
ACTIN	Reverse	CCAGAGGCGTACAGGGATAG
PLIN1	Forward	TGTGCAATGCCTATGAGAAGG
PLIN1	Reverse	AGGGCGGGGATCTTTTCCT
INSR	Forward	AAAACGAGGCCCGAAGATTTC
INSR	Reverse	GAGCCCATAGACCCGGAAG
COL5A3	Forward	TGACCGGGCATTCAGAATTGG
COL5A3	Reverse	CGGGCACCCCTTTCATCAT
EXOC7	Forward	AGCCCGGAACTCAACAAAGTG
EXOC7	Reverse	ATCAGGCTGCGAAATTCGGA
PCSK1	Forward	CTGGATGGCATTGTGACGGAT
PCSK1	Reverse	GCCCCAGCTTGCACTGTAAA
ABCA1	Forward	ACCCACCCTATGAACAACATGA
ABCA1	Reverse	GAGTCGGGTAACGGAAACAGG

Table S12: RT-qPCR differential expression to confirm knockdown.

Knockdown cell line	qPCR target	beta_actin ct mean	beta_actin ct SEM	target gene ct mean	target gene ct sem	delta_ct mean	delta_ct SEM	ddct mean	ddct SEM	ddct 95% CI lower bound	ddct 95% CI upper bound	FC	FC 95% CI lower bound	FC 95% CI upper bound	ddct pval (1-sided)
Cas9	ABCA1	16.773	0.084	26.103	0.268	9.330	0.281	0.000	0.281	-0.551	0.551	1.000	0.683	1.465	5.00e-01
ABCA1	ABCA1	14.550	0.026	26.673	0.195	12.123	0.196	2.793	0.196	2.408	3.178	0.144	0.110	0.188	1.55e-05
Cas9	PCSK1	18.613	0.050	27.633	0.171	9.020	0.178	0.000	0.178	-0.350	0.350	1.000	0.785	1.274	5.00e-01
PCSK1	PCSK1	17.767	0.093	27.467	0.368	9.700	0.379	0.680	0.379	-0.063	1.423	0.624	0.373	1.045	6.65e-02
Cas9	PPARG1	15.737	0.017	23.400	0.049	7.663	0.052	0.000	0.052	-0.102	0.102	1.000	0.932	1.073	5.00e-01
PPARG	PPARG1	15.303	0.098	23.503	0.061	8.200	0.116	0.537	0.116	0.310	0.763	0.689	0.589	0.807	2.82e-03
Cas9	PPARG2	15.737	0.017	27.173	0.239	11.437	0.239	0.000	0.239	-0.469	0.469	1.000	0.722	1.384	5.00e-01
PPARG	PPARG2	15.303	0.098	31.067	0.032	15.763	0.103	4.327	0.103	4.124	4.529	0.050	0.043	0.057	7.29e-08
Cas9	PLIN1	16.463	0.049	35.967	0.467	19.503	0.469	0.000	0.469	-0.920	0.920	1.000	0.528	1.892	5.00e-01
PLIN1	PLIN1	16.300	0.035	35.903	0.642	19.603	0.643	0.100	0.643	-1.159	1.359	0.933	0.390	2.234	4.41e-01

Table S13: Effect of knockdown on lipid accumulation.

BODIPY lipid accumulation 10x, differentiated

Checked for Cook's distance outliers (**one outlier removed for *SLTM***)

Two-sided tests (control vs. knockdown)

**Knockdown cell line**	**Fold change** (mean cytoplasm fluorescence, relative to Cas9 empty)	***t*-test sample size**	**Two-sided *t*-test pval**
Cas9 empty	1	-	-
hWAT	1.199	12	1.02E-01
INSR	1.115	12	4.32E-01
PPARG	0.245	12	5.52E-07
ABCA1	1.296	12	5.23E-02
COL5A3	1.723	12	2.78E-03
DENND5B	0.808	12	6.98E-02
EXOC7	1.354	12	9.63E-03
HERC1	1.149	12	2.18E-01
MFAP5	1.185	12	3.23E-01
MLXIPL	1.149	12	3.99E-01
SLTM	0.514	11	1.91E-04
TRIP10	1.386	12	1.57E-02
UBR2	1.23	12	2.29E-01

Table S14: Evidence of druggability for knockdown target genes.

Gene	**Effect of gene knockdown** in hWAT cells on lipid accumulation (fold change; P-value)	Known drugs	Tractability		
			**Small molecule**	**Antibody**	**PROTAC**
ABCA1	Non-significant<br>1.30 (0.052)	PROBUCOL - small molecule inhibitor	Structure with ligand<br>Druggable family	UniProt loc high conf<br>GO CC high conf<br>UniProt SigP or TMHMM	Half-life data
COL5A3	Increases lipid accumulation*<br>1.72 (0.0028)	COLLAGENASE CLOSTRIDIUM HISTOLYTICUM -<br>enzyme, acting through collagen hydrolytic enzyme mechanism<br>OCRIPLASMIN - protein, acting through collagen hydrolytic enzyme mechanism	-	UniProt loc high conf<br>GO CC high conf<br>UniProt loc med conf<br>UniProt SigP or TMHMM<br>Human Protein Atlas loc	-
DENND5B	Non-significant<br>0.81 (0.070)	-	-	UniProt SigP or TMHMM	Database ubiquitination<br>Half-life data
EXOC7	Increases lipid accumulation*<br>1.35 (0096)	-	-	UniProt loc high conf<br>GO CC high conf<br>UniProt loc med conf<br>Human protein atlas loc	Database ubiquitination<br>Half-life data
HERC1	Non-significant<br>1.15 (0.22)	-	Structure with ligand	UniProt loc med conf	Database ubiquitination<br>Half-life data
INSR	Non-significant<br>1.12 (0.43)	INSULIN - protein, insulin receptor agonist<br>LINSITINIB, BMS-754807, KW-2450 FREE BASE, KW-2450 - small molecule, insulin receptor inhibitors	Structure with ligand<br>High-quality ligand<br>High-quality pocket<br>Druggable family	GO CC high conf<br>UniProt loc med conf<br>UniProt SigP or TMHMM<br>Human protein atlas loc	Database ubiquitination<br>Half-life data<br>Small molecule binder
MFAP5	Non-significant<br>1.19 (0.32)	-	-	GO CC high conf<br>UniProt loc med conf<br>UniProt SigP or TMHMM	-
MLXIPL	Non-significant<br>1.15 (0.40)	-	Structure with ligand	-	-
PPARG	Reduces lipid accumulation**<br>0.25<br>(5.5×10-7)	PIOGLITAZONE HYDROCHLORIDE, MESALAMINE, TROGLITAZONE, ROSIGLITAZONE, PIOGLITAZONE, BALSALAZIDE, ROSIGLITAZONE MALEATE, BEZAFIBRATE, OLSALAZINE SODIUM, OLSALAZINE, BALSALAZIDE DISODIUM, RIVOGLITAZONE, LANIFIBRANOR, TESAGLITAZAR, IMIGLITAZAR, SAROGLITAZAR, MK-0767, MURAGLITAZAR, LERIGLITAZONE, CHIGLITAZAR, BARDOXOLONE METHYL, BALAGLITAZONE, ALEGLITAZAR, EFATUTAZONE, DB959, FARGLITAZAR, NAVEGLITAZAR, SODELGLITAZAR, ARHALOFENATE, ATX08-001, MK-0533, ETALOCIB, INT131, MBX-2044, INDEGLITAZAR - small molecule PPARG agonists	Structure with ligand<br>High-quality ligand<br>High-quality pocket<br>Druggable family	-	UniProt Ubiquitination<br>Database Ubiquitination<br>Small molecule binder
SLTM	Reduces lipid accumulation**<br>0.51 (1.9×10-4)	-	-	-	UniProt Ubiquitination<br>Database Ubiquitination<br>Half-life data<br>Small molecule binder
TRIP10	Increases lipid accumulation*<br>1.39 (0.016)	-	-	UniProt loc med conf	Database ubiquitination<br>Half-life data
UBR2	Non-significant<br>1.23 (0.23)	-	-	Human protein atlas loc	Database ubiquitination<br>Half-life data
